# Fabrication of Mie-resonant silicon nanoparticles using laser annealing for surface-enhanced fluorescence spectroscopy

**DOI:** 10.1038/s41378-024-00666-9

**Published:** 2024-03-28

**Authors:** Tatsuya Fukuta, Ryo Kato, Takuo Tanaka, Taka-aki Yano

**Affiliations:** 1 Institute of Post-LED Photonics, Tokushima University, 2-1 Minami-Josanjima, Tokushima, 770-8506 Japan; 2 Innovative Photon Manipulation Research Team, RIKEN Center for Advanced Photonics, Wako, Saitama, 351-0198 Japan; 3 Metamaterials Laboratory, RIKEN Cluster for Pioneering Research, Wako, Saitama, 351-0109 Japan

**Keywords:** Nanoparticles, Nanoparticles

## Abstract

Silicon nanostructures with unique Mie resonances have garnered considerable attention in the field of nanophotonics. Here, we present a simple and efficient method for the fabrication of silicon (Si) nanoparticle substrates using continuous-wave (CW) laser annealing. The resulting silicon nanoparticles exhibit Mie resonances in the visible region, and their resonant wavelengths can be precisely controlled. Notably, laser-annealed silicon nanoparticle substrates show a 60-fold enhancement in fluorescence. This tunable and fluorescence-enhancing silicon nanoparticle platform has tremendous potential for highly sensitive fluorescence sensing and biomedical imaging applications.

## Introduction

Dielectric nanostructures with high refractive indices and low optical losses have emerged as alternatives to plasmonic nanostructures, yielding a new trend in nanophotonics^1–3^. Unlike plasmonic nanostructures, high-index dielectric nanostructures exhibit simultaneous excitation of multipole Mie resonances. The interplay and interference between the electric and magnetic multipole Mie resonances induce directional scattering^4–6^, Huygens surfaces^7,8^, bound states in the continuum (BICs)^9^, and quasi-BICs^10,11^. The unique optical phenomena induced by dielectric nanoantennas and metasurfaces provide a wide spectral range of applications, including polarization-controlled optical devices^12,13^, highly sensitive optical biosensors^14–16^, and full-color printing^17–19^.

Although their enhancement factors are not as high as those of plasmonic nanostructures, dielectric nanostructures also exhibit significant field confinement and enhancement in their vicinity owing to their high refractive index. The field enhancement effect is significantly dependent on the Mie-resonant modes. Silicon (Si) is a promising high-refractive-index material that behaves as a dielectric at optical wavelengths due to its significantly low absorption. For instance, spherical Si nanoparticles with diameters of 100–200 nm exhibit greater field enhancement through magnetic dipole (MD) resonances than through electric dipole (ED), electric quadrupole (EQ), and magnetic quadrupole (MQ) resonant modes. This occurs because of the highest scattering efficiency and lowest extinction coefficient are produced at the MD resonant wavelengths. Multipole-induced enhancement of the incident field has been applied to surface-enhanced spectroscopies^20,21^. Surface-enhanced Raman spectroscopy has been demonstrated using various Si nanostructures, including nanoparticles^22,23^, nanogap antennas^24^, and nanodisk arrays^25^. Si-based dielectric metasurfaces with high-Q Mie resonances in the mid-infrared region have also enabled enhanced infrared absorption spectroscopy^26–28^. Furthermore, surface-enhanced fluorescence spectroscopy has benefited from dielectric nanostructures, as they prevent fluorescence quenching, which is unavoidable in metallic nanostructures^29^. Unlike plasmonic nanostructures, which require physical spacers to reduce the nonradiative decay of fluorophores near the metal surfaces^30–32^, dielectric nanostructures do not require these spacers; thus, the fluorescence enhancement becomes maximized when fluorophores are in direct contact with the dielectric surfaces. Another advantage of Si-based dielectric nanostructures in surface-enhanced spectroscopies is their significantly lower absorption compared to that of metals, enabling the suppression of optical heating, which is unavoidable in plasmonic nanostructures^24,33^. This allows for the minimally invasive detection of heat-sensitive biomolecules.

Among the high-index dielectric materials, Si is the most commonly used material for dielectric nanostructures. Spherical Si nanoparticles serve as the simplest and most ideal Mie resonators, constituting nanoantennas and metasurfaces and have been extensively studied in both fundamental research and practical applications. Various fabrication techniques have been developed for Si nanoparticles. One effective method for synthesizing Si nanoparticles with Mie resonances in the visible range is laser ablation^6,34–36^. Si-nanoparticle fragmentation is induced by irradiating a Si wafer with femtosecond pulses followed by random deposition near the focal point. Additionally, the laser printing technique allows precise positioning of Si nanoparticles by transferring the laser-ablated nanoparticles onto another closely facing substrate, enabling nanoscale control of the separation distance between the nanoparticles^37^. Laser ablation conducted in a liquid environment results in the formation of colloidal Si nanoparticles^38,39^. The bottom-up approaches based on laser-assisted methods have also been demonstrated. For instance, annealing SiO powders at temperatures above the melting point of Si initiates the disproportionation of SiO into Si, resulting in the formation of perfectly spherical Si nanoparticles^40,41^.

Herein, we present the practical fabrication of Mie-resonance-based Si-nanostructured substrates using continuous wave (CW) laser annealing. By focusing the light from a CW laser with a wavelength of 532 nm onto amorphous Si thin films, the formation of Si nanoparticles was induced over the laser focus area. These Si nanoparticles exhibited a homogeneous size distribution, enabling the uniform excitation of the Mie resonances in the visible region. We investigated the crucial role of the polymer underlayers in producing high-crystallinity Si nanoparticles under moderate incident laser power. The Mie resonances of the Si nanoparticle substrates provided a 60-fold enhancement in fluorescence when the nanoparticle surface was functionalized with fluorescent dye molecules. Importantly, the Mie resonant wavelengths of the Si nanoparticles could be controlled across the visible region by adjusting the laser annealing conditions, facilitating the utilization of various fluorescent dyes. Fluorescence-enhancing Si nanoparticle substrates with controlled Mie resonances have significant potential for enhanced fluorescence sensing and imaging of biomedical samples.

## Results and discussion

**Fig. 1:**
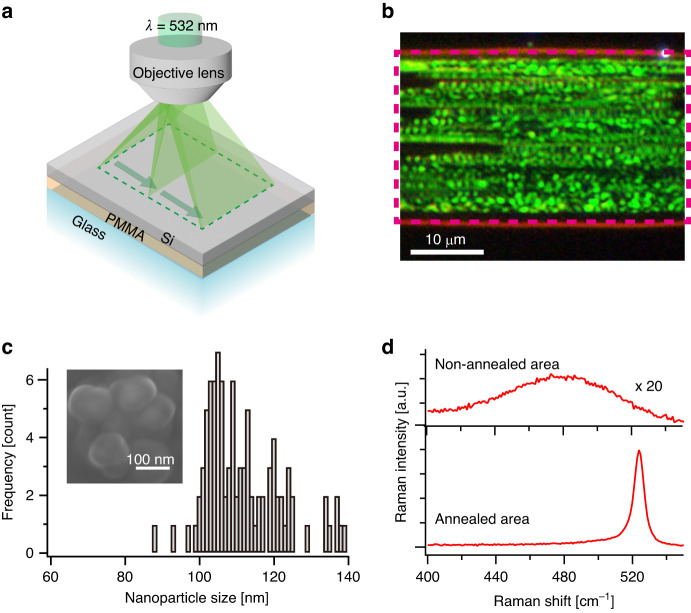
Laser annealing for fabrication of the Si nanoparticles. **a** Schematic illustration of the laser annealing process. Line-shaped CW laser light with a wavelength of 532 nm is focused onto a Si thin film deposited on a PMMA substrate. Laser-annealing is achieved by scanning the line-focused light along the orthogonal direction of the laser line. **b** Dark-field optical image of the laser-annealed Si thin film. **c** SEM image of the laser-annealed Si nanoparticles exhibiting an average diameter of 110 nm. **d** Raman spectra of Si nanoparticles: Si formed in the laser-annealed area and the as-deposited Si thin film in the nonannealed area

Laser annealing was performed by focusing line-shaped CW laser light with a wavelength of 532 nm onto an 80-nm-thick Si thin film deposited on a poly(methyl methacrylate) (PMMA) substrate. The laser power density and exposure time were set as 18 kW/cm^2^ and 0.1 s, respectively. As schematically illustrated in Fig. 1a, annealing of the Si thin film was achieved by scanning the line-focused laser along the orthogonal direction of the laser line. Figure 1b shows a dark-field optical image of a laser-annealed Si thin film. The laser-illuminated area exhibited dominant green scattering, whereas no significant optical scattering was observed in the nonilluminated area owing to the smooth and flat surface of the as-deposited Si thin film with a 0.22-nm roughness (see Fig. S1). Scanning electron microscopy (SEM) observation and analysis of the green dots in the dark-field optical image confirmed the formation of nanoparticles with an average diameter of 110 nm (Fig. 1c). The Raman spectrum of the nanoparticles (Fig. 1d) showed a strong peak at 520 cm^−1^, corresponding to the transverse optical (TO) phonon mode of crystalline Si. In contrast, the Raman spectrum of the as-deposited Si thin film exhibited a weak and broad peak at 472 cm^−1^, corresponding to the TO phonon mode of amorphous Si. The crystallinity of Si was evaluated from the Raman intensity ratio of the crystalline and amorphous TO modes^42,43^, revealing that the laser-annealed Si nanoparticles contained 92% crystalline phase and 8% amorphous phase. To gain insight into the crystalline nature of the Si nanoparticles, cross-sectional TEM measurements were performed. As depicted in Fig. S1c, the interior of a Si nanoparticle was closely observed. The diffraction pattern, predominantly from the (111) plane, as shown in Fig. S1d, confirmed the crystallization of the Si nanoparticles. Therefore, highly crystalline Si nanoparticles were formed by CW laser annealing.

To determine the extent of oxidation, XPS (Si 2p) was conducted on the topmost Si surface both before and after annealing. As shown in Fig. S1e, the results indicated significant SiO_2_-derived intensity on the surface. For a deeper perspective and due to the limited probing depth of XPS, FE-SEM was used. The FE-SEM image can be used to differentiate between Si and SiO_2,_ especially under high acceleration voltages, and is shown in Fig. S1f. As shown in the image, the SiO_2_ shell was approximately 5 nm thick and predominantly formed on the outermost surface of the Si nanoparticles.

**Fig. 2:**
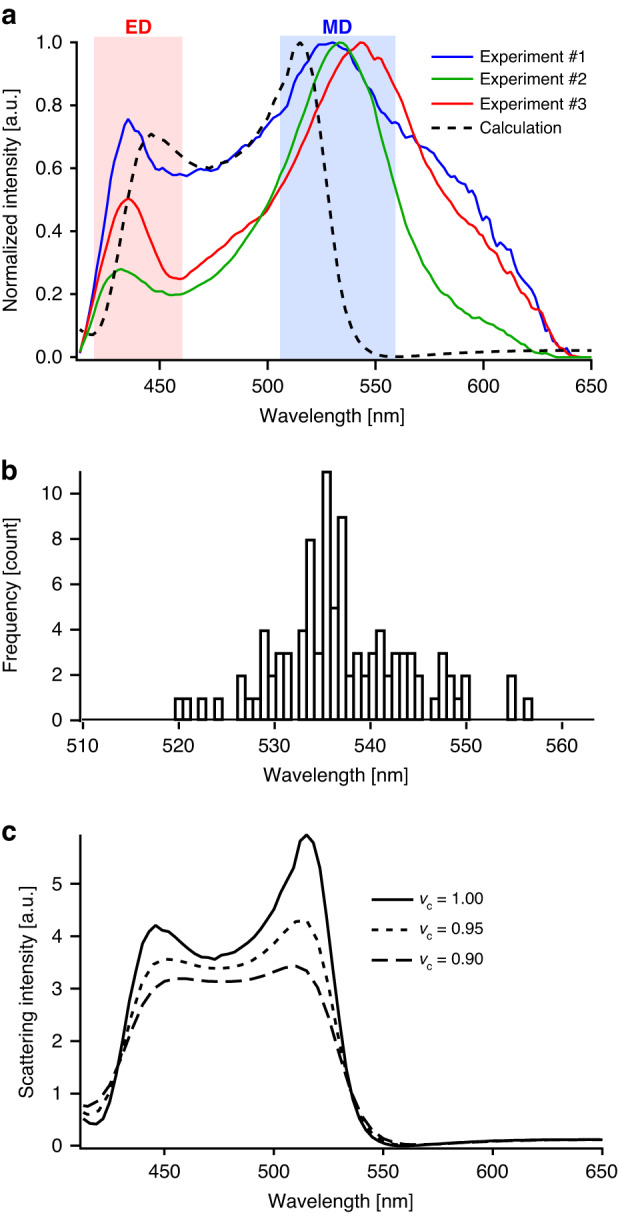
Mie resonances of the laser-annealed Si nanoparticles. **a** Dark-field scattering spectra of the Si nanoparticles with an average diameter of 110 nm. The spectra exhibit two dominant peaks at approximately 435 and 530 nm, corresponding to ED and MD modes, respectively. The dotted line is the calculated spectrum of a Si nanoparticle of the same diameter. **b** Wavelength distribution of the MD mode showing strong localization of approximately 535 nm. **c** Calculated scattering spectra of a nanoparticle composed of c-Si and a-Si with different volume fractions (*v*_*c*_ = 1.00, 0.95, and 0.90)

To investigate the optical resonances of the Si nanoparticles observed as green dots in the dark-field optical image, dark-field scattering spectra were measured in the backscattering configuration. Figure 2a shows the representative spectra of the Si nanoparticles with an average diameter of 110 nm. The spectra were decomposed into two dominant peaks at approximately 435 nm and 530 nm; these were attributed to the ED and MD modes of the silicon nanoparticles, respectively. As shown in the wavelength distribution of the MD mode (Fig. 2b), the MD modes were well localized at approximately 535 nm, which explained the green color of most of the laser-annealed Si nanoparticles in the dark-field image in Fig. 1b. However, the experimental scattering spectra deviated from the calculated spectra, as indicated by the dotted spectrum in Fig. 2a. The calculated scattering spectra of a crystalline Si nanoparticle with a diameter of 110 nm exhibited MD modes at 515 nm; this wavelength was shorter than the corresponding wavelength in the experimental spectra. A probable cause of the difference between the experimental and simulated spectra was the size distribution of the laser-annealed Si nanoparticles. In general, the resonant wavelengths of Si nanoparticles changed by several tens of nanometers with a mere 5-nm difference in particle size, resulting in a variation in the peak position in the experimental spectra. Another possible reason was the spherical geometric deviation of the laser-annealed Si nanoparticles. As shown in the SEM image in Fig. 1c, the nanoparticles were not perfectly spherical. The slightly distorted nanoparticles induced a small change in the resonant wavelength compared to that of spherical nanoparticles of the same size.

The crystallinity of Si nanoparticles is also crucial for determining the Mie resonance. The scattering spectra of the Si nanoparticles composed of crystalline silicon (c-Si) and amorphous silicon (a-Si) were numerically investigated via Raman spectroscopic analysis, as shown in Fig. 1c; the results revealed that the laser-annealed Si nanoparticles were not perfectly crystallized and contained ~8% a-Si. To calculate the scattering spectra of the Si composites, effective dielectric functions were deduced from Bruggeman’s effective medium approximation^42^ by solving Eq. (1).

1 $${\upsilon }_{a}\frac{{\epsilon }_{a}-{\epsilon }_{\rm{eff}}}{{\epsilon }_{a}-{2\epsilon }_{\rm{eff}}}+{\upsilon }_{c}\frac{{\epsilon }_{c}-{\epsilon }_{\rm{eff}}}{{\epsilon }_{c}-{2\epsilon }_{\rm{eff}}}=0\,{\rm{and}}\,{\upsilon }_{a}+{\upsilon }_{c}=1$$

where *ε*_*a*_ and *ε*_*c*_ are the dielectric functions of *a*-Si and *c*-Si, respectively. *ε*_eff_ is the effective dielectric function of the Si composite. *v*_*a*_ and *v*_*c*_ are the volume fractions of *a*-Si and *c*-Si, respectively. Figure 2c shows the calculated scattering spectra of the Si nanoparticle composites with different volume fractions (*v*_*c*_ = 1.0, 0.95, and 0.90). As *v*_*a*_ increased, the ED and MD modes were slightly redshifted and blueshifted, respectively. More prominently, the scattering intensity ratio between the ED and MD modes significantly varied with a very small change in volume fraction, resulting in a variation in the intensity ratio observed in the experimental spectra.

Notably, Si nanoparticles were formed when a polymer (PMMA) substrate was used as an underlayer for the Si thin film. To elucidate the effects of the underlayer on the laser annealing of the Si thin films, laser annealing was performed under the same conditions as those for which the thin films were directly deposited on a glass substrate without the PMMA buffer layer. As shown in Fig. S2a, the dark-field optical scattering image of the Si thin film did not exhibit any changes in contrast or color between the laser-illuminated and unilluminated areas. Furthermore, the Raman spectrum of the Si thin film confirmed the presence of an amorphous phase even after laser annealing (see Fig. S2b), indicating that the crystalline Si nanoparticles were not formed without a PMMA underlayer. The crucial difference between the two underlayer materials (PMMA and glass) during laser annealing was their thermal conductivities. The thermal conductivity of a 50-nm-thick PMMA thin film is 0.1 W/mK, while that of a glass slip is 1 W/mK. The thermal conductivity of PMMA is one order of magnitude lower than that of glass, which hinders heat diffusion from the optically heated Si thin film into the underlying material. Consequently, the Si thin film on the PMMA underlayer experienced significant heating at the laser focus spot, inducing the formation of nanoparticles. This scenario was further validated experimentally using other materials as underlayers with different thermal conductivities (*K*). Crystalline Si nanoparticles were formed from polystyrene thin films (*K* = 0.12 W/mK), which possessed conductivities close to those of PMMA, whereas no crystalline Si nanoparticles were formed from indium tin oxide (*K* = 4 W/mK) and aluminum (*K* = 205 W/mK), which possessed considerably greater thermal conductivities than glass. Notably, the underlying layers were exposed to high temperatures exceeding 500 °C directly beneath the laser-heated Si substrate, as thermal crystallization of amorphous materials typically requires elevated temperatures. For the PMMA underlayer, SEM-based energy dispersive spectroscopy (EDS) revealed a significant reduction in carbon content attributed to PMMA (Fig. S2c) after laser annealing, which indicated that the polymer layer was heated to an elevated temperature and partially vaporized.

During the process of laser annealing for crystallizing amorphous Si nanoparticles, the duration of laser exposure is also a pivotal factor in the crystallization process. While elevated temperatures expedite the kinetics of crystallization, the exact dynamics between temperature and exposure time can be complex and depend on several factors, including nanoparticle size, substrate material, and ambient conditions. According to our methodology using CW laser annealing, the temperature rapidly increased, initiating rapid crystallization of the amorphous silicon. This brief yet intense temperature spike ensured that the formed Si nanoparticles achieved the desired crystallinity. For our experiments, we set the exposure time at a consistent 0.1 s. This timeframe proved adequate for the crystallization of the a-Si nanoparticles under our chosen conditions. Notably, increasing the exposure time did not significantly enhance the crystallinity. However, prolonged exposure led to an increase in nanoparticle size. For instance, when the exposure time approached 1 s, the resultant nanoparticles became large enough to cause a shift in the Mie-resonant wavelength and shifted it out of the visible range. Due to these observations, an exposure time of 0.1 s was used as the optimized time to create a balance between achieving the desired crystallinity and controlling nanoparticle size.

In addition to the use of laser annealing for the fabrication of Mie resonance-based Si nanoparticles, the application of laser annealing to Si thin-film transistors and solar cells has been extensively attempted, in which the crystallization of an amorphous Si layer through laser annealing contributes to enhanced device performance. Pulsed excimer lasers with a wavelength of 304 nm have been widely used for laser annealing because of the strong optical absorption of Si, which enables effective optical heating-induced crystallization. Laser-induced annealing has also been demonstrated using CW visible lasers, which are more readily available and cost-effective than excimer lasers because they do not require the exchange of toxic gases used in excimer lasers. The CW-laser wavelength of 532 nm used in our experiments was not as closely matched to the absorption peak of amorphous Si as that of excimer lasers but still fell within the absorption window, resulting in moderate crystallization of amorphous Si. Additionally, the cooling rate induced by the CW laser was much lower than that induced by pulsed lasers, initiating the formation of larger Si grains/nanoparticles with diameters of hundreds of nanometers.

**Fig. 3:**
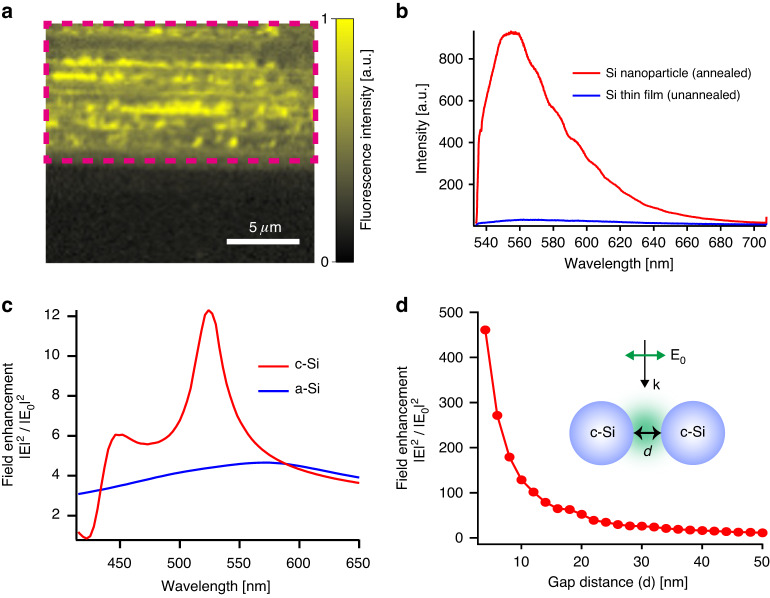
Near-field intensity enhancement of the Si nanoparticles. **a** Fluorescence image of the Si substrate obtained at the emission of 550–560 nm using an excitation wavelength of 532 nm. The laser-annealed area is indicated by the dotted rectangle. **b** Fluorescence spectra measured on the annealed and unannealed areas. The laser-annealed Si nanoparticles provide more than a 60-fold stronger fluorescence intensity than the unannealed Si thin film substrate. **c** Calculated field enhancement at the outmost surfaces of a-Si and c-Si nanoparticles with a diameter of 110 nm. **d** Calculated field enhancement at the gap between two c-Si nanoparticles plotted as a function of the gap distance

Mie-resonant Si nanoparticles were used for field-enhanced fluorescence microscopy. The surface of the laser-annealed Si substrate was chemically functionalized with fluorescent dye molecules (Alexa Fluor: *λ*_ex_ = 532 nm, *λ*_em_ = 554 nm). Figure 3a shows a fluorescence image of the Si substrate obtained at an emission wavelength ranging from 550–560 nm at an excitation wavelength of 532 nm. Strong fluorescence was predominantly observed over the laser-annealed area, indicated by the dotted rectangle in Fig. 3a, whereas the remaining unannealed area corresponding to the amorphous Si thin film exhibited weak fluorescence. The corresponding dark-field scattering image of the annealed area provided an intensity contrast similar to that of the fluorescence image (data not shown), indicating fluorescence enhancement near the Si nanoparticles. Figure 3b shows the fluorescence spectra of the annealed and unannealed regions. The Si nanoparticle substrate provided more than 60 times stronger fluorescence intensity than the Si thin-film substrate at the maximum emission wavelength of 554 nm. This high fluorescence enhancement was attributed to the resonant excitation of the MD mode of Si nanoparticles with a diameter of 110 nm. As inferred from Fig. 2a, the Si nanoparticles exhibited an MD mode at approximately 535 nm, which closely matched the excitation wavelength of the fluorescent dye molecules. The MD mode of the nanoparticles was strongly localized at the outermost surface as well as inside the particle, enabling Mie-resonant excitation of the dye molecules functionalized on the nanoparticle surface.

The crystallization of Si nanoparticles played another crucial role in achieving a high near-field enhancement of the MD mode. Figure 3c shows the simulated near-field spectra at the surfaces of the amorphous and crystalline Si nanoparticles with a diameter of 110 nm. The results demonstrated that the strongest near-field enhancement was obtained in the MD mode for crystalline Si nanoparticles. Conversely, the near-field enhancement significantly decreased for the amorphous Si nanoparticles. This difference was attributed to the extinction coefficient of amorphous Si being one order of magnitude larger than that of crystalline Si. This higher extinction coefficient in amorphous Si led to the dampening of the Mie resonance, thus suppressing the field enhancement.

To comprehensively understand the observed fluorescence enhancement, we needed to consider the interplay between neighboring nanoparticles, as individual field enhancement from a single Si nanoparticle was not adequate to explain more than 60 times the enhancement in fluorescence. Specifically, the separation distance between these nanoparticles, as evident from the SEM image in Fig. 1c, ranged from 0 to a few tens of nanometers. Figure 3d shows the near-field intensity enhancement at the gap between two crystalline Si nanoparticles as a function of the separation distance. As the gap distance decreased, the near-field intensity increased, mirroring the behavior of plasmonic dimers. Remarkably, the enhancement reached more than 100 times at distances less than 10 nm. Notably, the near-field resonant wavelength, where the maximum near-field intensity of the MD mode was observed at the gap, remained consistent across the different separation distances; this result was different than observations with plasmonic nanoparticles.

**Fig. 4:**
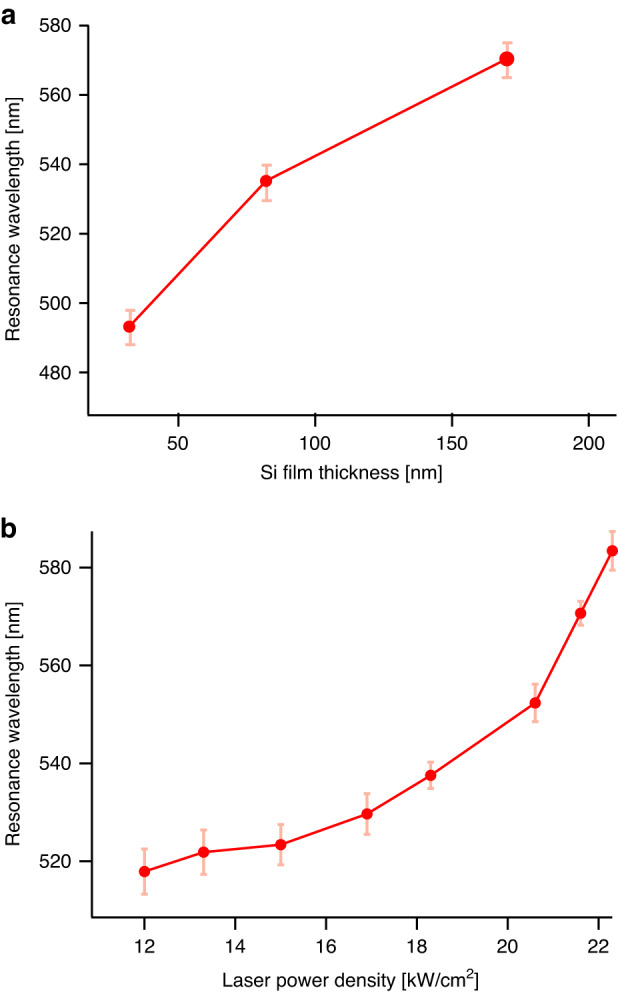
Tunability of the Mie-resonant wavelengths in the Si Nanoparticles. **a** Si film thickness dependence of the MD mode wavelength, shows a redshift from 484 to 560 nm as the film thickness increases from 30 to 170 nm. The laser power density was kept constant at 18 kW/cm^2^. Insets depict typical SEM images of the Si nanoparticles corresponding to the various film thicknesses, with a 100 nm scale bar. **b** Laser-power-density dependence of the MD mode wavelength, exhibiting an evident redshift with increasing laser power density. The Si film thickness was kept constant at 80 nm for all measurements

Finally, to apply Si nanoparticle-enhanced fluorescence spectroscopy to other visible wavelengths, we demonstrated the control of the Mie-resonant wavelength of the laser-annealed Si nanoparticles in the visible region. Among the various parameters for the laser annealing of amorphous Si thin films, the thickness of the Si films and the laser power density were found to be crucial factors affecting the sizes of the nanoparticles, enabling the tuning of the resonant wavelength of the MD mode. Figure 4 illustrates the influence of these laser annealing parameters on the resonant wavelengths. As the film thickness increased from 30 to 170 nm, the MD mode redshifted from 484 to 570 nm. This shift was attributed to the variation in the nanoparticle diameter, which changed from 80 to 153 nm, as evidenced by the SEM observations. Furthermore, as shown in Fig. 4b, the laser power density exhibited an evident redshift of the MD mode with increasing power density. Although these parameters did not show a complete linear dependence on the resonant wavelengths, the combination of their values enabled the tuning of the MD modes from approximately 480 to 630 nm. The tunable range of resonant wavelengths covered the excitation wavelengths of a large variety of commercially available fluorescent dyes, facilitating the practical application of highly sensitive fluorescence spectroscopy using laser-annealed Si nanoparticles.

While the inhomogeneity observed in the laser-annealed Si substrates (as shown in Figs. 1b and 3a) occurs primarily from the variations in the nanoparticle size and distribution, the imaging of smaller biosamples could be affected. For samples such as cells that span several tens of micrometers, the uneven optical field could mask or hinder the biological contrast. However, for larger biosamples, such as tissues encompassing several hundred micrometers, this inhomogeneity becomes less prominent, causing a less significant impact on imaging applications. We recognize the need and the importance of further refining the surface homogeneity for diverse bioimaging applications, especially as we progress toward exploring a broader range of biosamples using these substrates.

The distinctive merits of our CW laser annealing technique in the context of fabricating Mie-resonant Si nanostructures need to be highlighted. In stark contrast to the point-by-point writing process of EB lithography, our method allows for swift coverage of extensive areas, enhancing both processing speed and throughput. This advantage is coupled with significant cost efficiency, a critical factor for large-scale production. In comparison to pulsed laser annealing, which tends to produce smaller nanoparticles due to rapid cooling, our CW laser annealing facilitates the growth of larger Si grains/nanoparticles. These larger particles are essential for achieving effective Mie resonance in the visible spectrum. Due to the ability to control the particle size, combined with the method’s speed and cost-effectiveness, our technique has a significant advantage over other EB- and laser-based techniques, providing a more efficient, economical, and versatile solution for nanoparticle fabrication.

In conclusion, the Mie-resonant Si nanostructured substrates were successfully fabricated using CW laser annealing. The use of a PMMA underlayer during laser annealing played a crucial role in inducing the formation of crystalline Si nanoparticles, which exhibited efficient excitation of the MD mode and significant near-field enhancement. We achieved a 60-fold enhancement in the fluorescence intensity in the vicinity of the Si nanoparticles; this enhancement was attributed to the resonant excitation of the MD mode and the strong localization of the near field at the nanoparticle surfaces. Furthermore, we demonstrated the tunability of the Mie-resonant wavelengths in the visible region, enabling the practical application of Si nanoparticle-enhanced fluorescence spectroscopy at various wavelengths. Our proposed technique facilitates new possibilities for highly sensitive fluorescence-based applications, including optical sensing and imaging. Our findings provide valuable insights for the design and fabrication of dielectric nanoantennas and metasurfaces for advanced nanophotonic devices and systems.

## Materials and methods

### Reagents and chemicals

All chemicals and reagents used in this study were analytical grade and were used as received without further purification. Alexa Fluor 532 NHS ester, (3-aminopropyl)triethoxysilane (APTES), and poly(methyl methacrylate) (PMMA; average molecular weight of 15,000) were purchased from Thermo Fisher Scientific, Tokyo Chemical Industry Co., Ltd. (TCI), and Sigma-Aldrich, respectively. The Si evaporation target (99.9999%) and dimethyl sulfoxide (DMSO) were purchased from NewMet, Ltd., and Fujifilm (Wako Pure Chemicals), respectively.

### Fabrication of the Si/PMMA/glass substrates

Glass slips were thoroughly cleaned by ultrasonication three times at 40 °C for 20 min in ethanol, rinsed thoroughly with ultrapure water, and dried under a stream of nitrogen gas. The PMMA thin films were coated onto glass substrates by spin-casting a 2 wt% PMMA/toluene solution at a rate of 1500 rpm, resulting in a film thickness of 50 nm. The PMMA-cast glass substrates were further coated with 80-nm-thick Si thin films by electron-beam physical vapor deposition (deposition rate: 0.11 nm/s, vacuum pressure: 1.9 × 10^−5^ Pa).

### Laser annealing of the Si thin films

Laser annealing was performed by focusing a line-shaped CW laser beam (*λ* = 532 nm) onto the Si thin films using an objective lens (50×, 0.45 NA). To shape the beam, a cylindrical lens was used to expand the point source of the laser into a line shape. After collimation, a uniform intensity region of this line, approximately the central fifth of the beam, was focused on the sample surface. This methodology ensured that the laser light when projected as a line on the sample surface, had a uniform intensity distribution with an RMS variation of approximately 3%.

The laser beam, with a line length of approximately 100 µm, was directed using a Galvano scanner. Instead of undergoing continuous scanning motion, the Galvano scanner moved the beam perpendicular to the laser line with a step of 400 nm, while the mirror remained static during the laser irradiation at each step. After the exposure time of 0.1 s, the galvano mirror moved to the next irradiation position at a speed of 200 nm/ms. This specific operation, combined with shutter-controlled laser light, ensured that the laser was active only during the exposure time. These procedures enabled the achievement of laser annealing over an area of 100 µm × 100 µm in 1 min.

The laser power density was calculated from the size of the focused line-shaped laser beam, which was measured from the beam profile.

### Functionalization of the fluorescent dye on the laser-annealed Si substrates

APTES ethanolic solution (0.2 ml, 0.2 wt%) was drop-cast on the laser-annealed Si substrates and left for 30 min in a sealed box to avoid evaporation of the solvent. The substrate was subsequently rinsed with ultrapure water and baked at 100 °C for 15 min. Finally, 0.2 ml of the Alexa solution (0.5 wt%) was drop-cast onto the APTES-functionalized Si substrates and left for 30 min, and the dye-functionalized substrates were then rinsed with ultrapure water.

### Morphological and elemental characterization

The laser-annealed Si substrates were coated smoothly with a 1.0-nm-thick osmium layer to produce conductive substrates, and the coated substrates were then observed via field-emission SEM (Phenom Pharos, Thermo Scientific) equipped with EDS. Secondary SEM images and EDS spectra were obtained at an accelerating voltage of 10 kV. The surface roughness of the Si substrates was measured using AFM (AFM5500M, Hitachi High-Tech) in tapping mode.

### Optical characterization

Raman spectroscopic analysis of the crystallinity of the laser-annealed Si surfaces was performed using a slit-scanning confocal Raman microscope (Raman11, Nanophoton) with an excitation wavelength of 532 nm and an objective lens with an NA of 0.45. Fluorescence spectral imaging of the dye-functionalized Si substrates was performed using the same microscopic system. The laser power densities for the Raman and fluorescence measurements were set to 1.97 and 0.11 kW/cm^2^, respectively, both of which were sufficiently low to avoid laser annealing of Si. Mie-scattering images and spectra were obtained using an in-house microspectroscopic system combined with a dark-field inverted optical microscope (BX51, Olympus) and a self-scanning hyperspectral camera (NH-TY6, EBA Japan).

### Electromagnetic simulation

COMSOL Multiphysics software (version 6.0) was used to perform a finite element analysis of the scattering spectra of the Si nanoparticles. In the calculations, single or dimer spherical Si nanoparticles were placed on a flat glass substrate with surrounding air media. The backward scattering spectra were calculated by considering the experimental conditions (the direction of the incident plane wave and the collection direction of the scattering wave). The dielectric constant of Si was obtained from ref. ^44^ for crystalline Si and ref. ^45^ for amorphous Si.

### Supplementary information

Supplemental Material
