# Supplementary material for: Fabrication of Mie-resonant silicon nanoparticles using laser annealing for surface-enhanced fluorescence spectroscopy
Source: Microsyst Nanoeng. 2024 Mar 28;10:45. doi: 10.1038/s41378-024-00666-9 (PMC10978982; doi:10.1038/s41378-024-00666-9)
Supplement: Supplementary file 1 — Supplemental Material [file 41378_2024_666_MOESM1_ESM.docx]

**Supporting Information**

Fabrication of Mie-resonant silicon nanoparticles using laser annealing for surface-enhanced fluorescence spectroscopy

Tatsuya Fukuta^1,2,3^, Ryo Kato^1,2,3^, Takuo Tanaka^1,2,3^*, and Taka-aki Yano^1,2,3^*

^1^Institute of Post-LED Photonics, Tokushima University, 2-1 Minami-Josanjima, Tokushima 770-8506, Japan

^2^Innovative Photon Manipulation Research Team, RIKEN Center for Advanced Photonics, Wako, Saitama 351-0198, Japan

^3^Metamaterials Laboratory, RIKEN Cluster for Pioneering Research, Wako, Saitama 351-0109, Japan

*Correspondence:

Taka-aki Yano, yano.takaaki@tokushima-u.ac.jp

Takuo Tanaka, t-tanaka@riken.jp


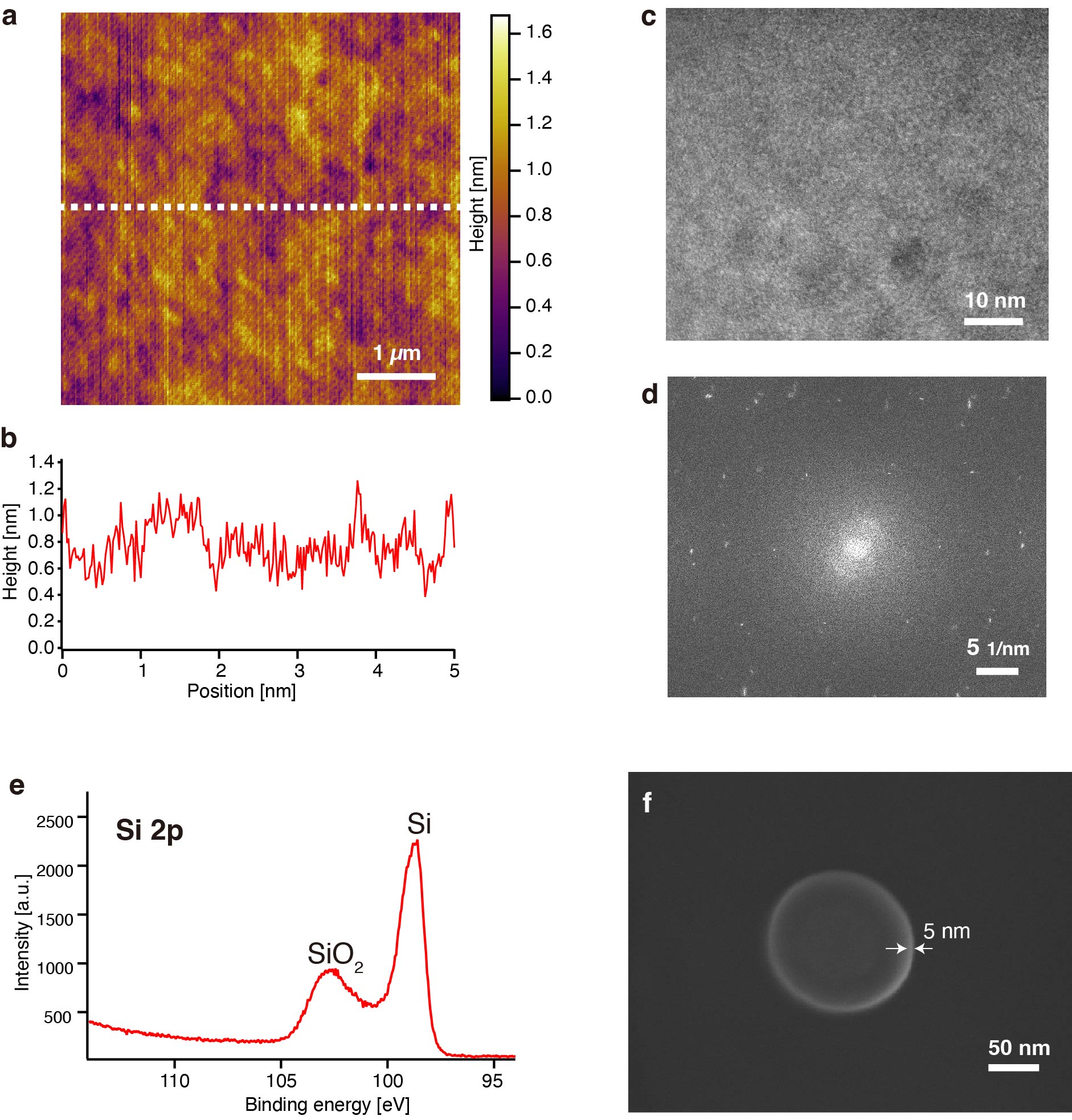


**Fig. S1** **a** Atomic force microscope (AFM) image of an as-deposited amorphous Si thin film. **b** Surface profile at the dotted line of a. **c** Cross sectional TEM image of a laser-annealed Si nanoparticle. **d** Diffraction pattern of c. **e** XPS spectrum (Si 2p) of the laser-annealed Si surface. **f** SEM image of a laser-annealed Si nanoparticle obtained using an acceleration voltage of 15 kV, showing the SiO2 oxidation shell as a bright contrast.


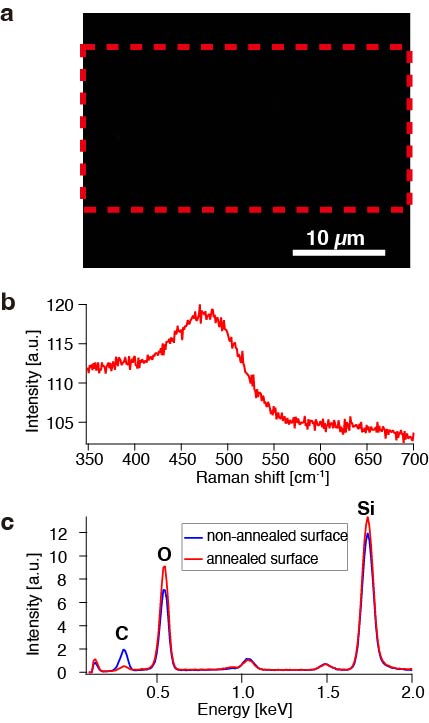


**Fig. S2** **a** Dark-field optical scattering image of a Si thin film directly deposited on a glass substrate without the PMMA buffer layer. **b** Raman spectrum of the Si thin film without the PMMA layer after the laser annealing process. **c** EDS spectrum of the Si thin film with the PMMA layer after the laser annealing process.
